# Lymph node micrometastases are associated with disease recurrence and poor survival for early-stage non-small cell lung cancer patients: a meta-analysis

**DOI:** 10.1186/s13019-016-0427-x

**Published:** 2016-02-16

**Authors:** Xu Feng Deng, Li Jiang, Quan Xing Liu, Dong Zhou, Bing Hou, Kefan Cui, Jia Xin Min, Ji Gang Dai

**Affiliations:** Department of Thoracic Surgery, Xinqiao Hospital, Third Military Medical University, Chongqing, 400037 China

**Keywords:** Non–small-cell lung cancer, Lymph node micrometastases, Meta-analysis

## Abstract

**Background:**

We performed a meta-analysis to clarify whether the molecular detection of tumor cells or micrometastases in the lymph node (LN) indicates a high risk of disease recurrence and poor survival in negative pathologic lymph node status non-small cell lung cancer (NSCLC).

**Methods:**

A literature search was performed using relevant keywords. We searched relevant studies from PubMed, Embase, and the Cochrane Library. Direct and indirect meta-estimates were generated using Review Manager software with fixed effects for the study. Study-to-study heterogeneity was summarized using *I*^2^ statistics and predictive intervals (PIs).

**Results:**

Our analysis of eight eligible studies revealed that patients with lymph node micrometastases (LNMM) were associated with poor overall survival (OS) (HR, 1.98; 95 % CI, 1.50 to 2.62; *p* < 0.00001) and disease-free survival (DFS) (HR, 2.34; 95 % CI, 1.67-3.27; *p* < 0.00001).

**Conclusion:**

LNMM is associated with an increased risk of disease recurrence and poor survival in patients with negative pathologic node negative NSCLC. Thus, these patients need to be carefully followed up after the initial pulmonary resection.

## Background

Lung cancer is the leading cause of cancer deaths worldwide, constituting 18 % of all cancer-related deaths. Up to 80–85 % of lung cancers are classified as non–small-cell lung cancer (NSCLC) [1]. Surgical treatment is an important therapy for early-stage non-small cell lung cancer (NSCLC). The extent of disease in NSCLC is staged according to the TNM classification [2], and because the stage at diagnosis is the principal prognostic indicator, accurate staging is essential for treatment decisions.

In theory, patients with early-stage non-small cell lung cancer can be cured by surgical resection alone. However, approximately 30 % of patients with pathologic stage 1 NSCLC have a recurrence of the tumor and die, despite complete surgical resection [3, 4]. This poor prognosis suggests the occurrence of micrometastasis, which often occurs before the primary operation and therefore cannot be assessed by routine clinical examinations and conventional histopathologic methods after the cancer has already spread to the regional lymph nodes. The detection of occult disease could help to identify those patients with lymph nodes micrometastases NSCLC who are at high risk for tumor recurrence and who might benefit from adjuvant therapy. Therefore, for an accurate prediction of prognosis, it is necessary to assess the lymph node status and to characterize micrometastasis.

The prognostic value of molecular tumor cell detection in patients with lymph node micrometastases NSCLC remains uncertain. To clarify this issue, we conducted a systematic review with meta-analyses of studies that evaluated the prognostic significance of molecular tumor cell detection in lymph nodes.

## Methods

### Search strategy

We performed a systematic search to identify articles published after 1996 that described the association between lymph node micrometastases of NSCLC and postoperative survival. Publications were included if they provided the most up-to-date analysis of at least one of the above-mentioned end points of interest. A literature search of Medline was performed using PubMed to identify published studies using the search terms (“lymph nodes micrometastases” OR “micrometastases”) AND (“non–small-cell lung cancer” OR “adenocarcinoma” OR “lung cancer”).

Before the beginning of this study, a rigorous protocol was developed according to the recommendations of the Cochrane Collaboration. The content of the documents identified by the search were then scrutinized by two researchers to determine eligibility for inclusion in the meta-analysis. All documents were included in our meta-analysis that evaluated the association of LNMM with disease-free survival (DFS) and overall survival (OS) of patients with negative pathologic node-negative NSCLC. The exclusion criteria were as follows: case report, clinical trials, letters to the editor and reviews without evaluation of the association of LNMM with overall survival (OS), and disease-free survival (DFS) of patients. Data from the included documents were entered into an electronic spreadsheet for analysis.

### Statistical analysis

Synchronized extraction results were pooled statistically as effect estimates in meta-analyses. HRs and their corresponding SEs were extracted for individual time-to-event outcome parameters of each primary study. If an HR and the associated SE or CIs were not reported, we approximated the HR by using the statistical data provided in the article [5, 6].

The extracted HRs were pooled by using the generic inverse-variance method available in the Review Manager software 5.2.9 (Cochrane Collaboration, Oxford, UK). Statistical heterogeneity among the trials was evaluated using Cochran’s Q test (*χ*^2^ test) and the Higgins I^2^ statistic to determine the percentage of total variation among studies resulting from heterogeneity. If the I^2^ statistic was ≤ 50 % the fixed effect model was used to pool studies; if not, the random effects model was used. Publication bias was assessed with an Egger’s regression intercept [7].

## Results

### Search results

**Fig. 1:**
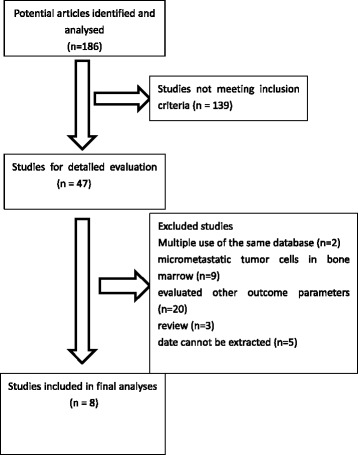
Selection diagram for studies

Eight eligible articles were analyzed [8–15]. All included documents were published between 1996 and 2011 (Tables 1 and 2). The details of the search are provided in Fig. 1.

**Table 1:** The basic information about the included studies

References	Definition of tumor-cell positivity	Detection method	Target gene/antigen	Follow-up (months)		Outcomes reported	stage	Multivariate analysis
				Median	Range			
Chun-Dong Gu	LNMM	IHC	CK(AbAE1/A)	44	4 –60	OS	I	Yes
Kazuhito Dobashi	LNMM	IHC	CK(AbAE1/A)	13.6	1-60	OS	pN0	Yes
B. Passlick	LNMM	IHC	CK	NR	NR	OS	pN0	Yes
Jakob R. Izbicki,	LNMM	IHC/FCM	Ber-Ep-4	NR	3-40	DFS	pN0	Yes
By Toshihiro Osaki	LNMM	IHC	CK(AbAE1/A)	35.8	0.1-90.6	OS	I	Yes
Kosei Yasumoto	LNMM	IHC	CK2	48.2	1-64	OS/DFS	I	Yes
Valerie W. Rusch	LNMM	IHC	CK(AE-1)	NR	0-60	OS/DFS	pN0	Yes
Wang Zhou	LNMM	RT-PCR	MUC1	NR	0-36	OS	pN0	Yes

*Abbreviations*: *LMMM* lymph node micrometastases, *IHC* immunohistochemistry, *CK* cytokeratin, *DFS* disease- free survival, *OS* overall survival, *NR* not reported, *RT-PCR* Reverse transcription-polymerase chain reaction. MUC1, polymorphic epithelial mucin 1

**Table 2:** Demographic data

Author	Year	LNM+	LNM-	HR(LNM+ VS LNM-)	95 %CI(Lower-upper)	In(HR)	SE(InHR)	Outcomes reported
Chun-Dong Gu	2002	22	27	2.12	0.53-8.41	0.75	0.7	OS
Kazuhito Dobashi	1997	14	17	5.1	0.61-42.66	1.63	1.08	OS
B. Passlick	2001	27	125	2.5	1.4-4.6	0.9	0.3	OS
Jakob R. Izbicki	1996	16	51	4.96	1.65-14.87	1.6	0.56	DFS
By Toshihiro Osaki	2002	32	83	1.7	0.57-5.09	0.53	0.56	OS
Kosei Yasumoto	2003	34	182	2.77	0.94-8.17	1.02	0.55	OS
Kosei Yasumoto	2003	34	180	3.06	1.38-6.78	1.12	0.41	DFS
Wang Zhou	2003	16	42	1.84	0.68-4.93	0.61	0.5	OS
Valerie W. Rusch	2011	130	450	1.63	1.13-2.36	0.49	0.19	DFS
Valerie W. Rusch	2011	130	450	1.59	1.13-2.23	0.46	0.17	OS

### Impact of LNMM status on OS

**Fig. 2:**
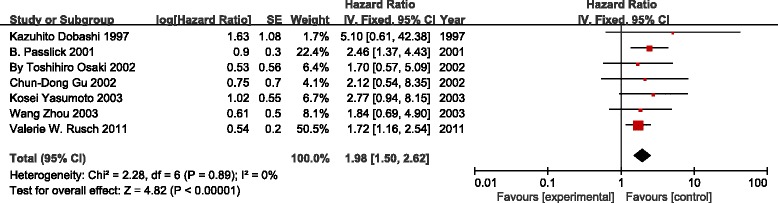
Meta-analyses on the association of molecular tumor cell detection in regional lymph nodes with overall survival

The impact of LNMM status on OS was assessed with the generic inverse-variance method available in the Review Manager software in five documents. The analysis identified a significantly different positive correlation between the presence of isolated tumor cells in LN at the time of diagnosis and a shortened overall survival duration (HR, 1.98; 95 % CI, 1.50 to 2.62; *p* < 0.00001). There was no evidence of statistical heterogeneity (I^2^ = 0 %, *χ*^2^ = 2.28, df = 6, *P* = 0.89) (Fig. 2).

### Impact of LNMM status on DFS

**Fig. 3:**
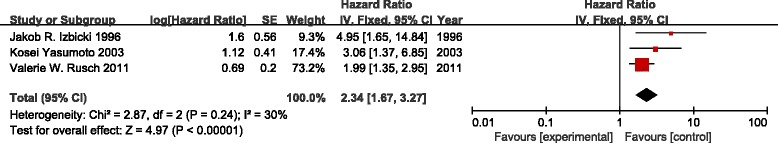
Meta-analysis on the association of molecular tumor cell detection in regional lymph nodes with disease-free survival

Only three documents reported the incidence of disease-free recurrence. Meta-analyses confirmed the prognostic significance of molecular tumor-cell detection in LN for the outcomes of DFS (HR, 2.34; 95 % CI, 1.67-3.27; *p* < 0.00001). Statistical heterogeneity was not detected (I^2^ = 30 %, *χ*^2^ = 2.87, df = 2, *P* = 0.24) (Fig. 3).

## Discussion

This systematic review and meta-analysis showed that the molecular detection of tumor cells in regional lymph nodes is associated with an increased risk of disease recurrence and poor survival in patients with negative pathologic lymph node status NSCLC.

Lung cancer remains the most common cancer in the world, both in terms of new cases (1.8 million cases, 12.9 % of all cancers) and deaths (1.6 million deaths, 19.4 %) because of the high number of fatal cases [16]. The TNM staging system of lung cancer is widely used as a guide for predicting the prognosis. The presence of lymph node metastases along with T and M status represents the most accurate factor that is currently available for the prediction of prognosis in patients who undergo complete surgical resection. However, a significant proportion of patients with early stage NSCLC who undergo potentially curative resections subsequently relapse and die. This suggests that occult micrometastasis can exist at the time of surgery; the rate is clearly underestimated by current clinical staging examinations and conventional histopathologic methods. A nodal micrometastasis was defined according to the definition of the International Union Against Cancer (UICC; i.e. as a single or small number of tumor cells that are 0.2 mm in dimension [ITCs] and as tumor deposits < 2.0 mm but greater than 0.2 mm in dimension [MMs]) [17]. If the UICC definition was not applied or mentioned explicitly, single or clustered cells that were detected in molecular analyses were also considered as occult disease. In this study, we identified occult micrometastatic tumor cells in pN0 lymph nodes in half of the patients with completely resected stage 1 NSCLC using an immunohistochemical staining assay. The patients with lymph node micrometastasis had a poorer prognosis than the patients without micrometastasis by univariate or multivariate analyses; the prognostic impact was independent from the TNM staging system [8].

For this meta-analysis, we attempted to closely follow the recommendations presented by the Cochrane Collaboration. We pre-specified a strict study protocol, and we searched documents from several international conferences, electronic databases and reference research for relevant trials. The language was not restricted. Eight documents, which were published worldwide from 1996 to 2011, were included in the meta-analysis. Despite the number of documents that have been reported and the well-explained risk of patients with LNMM, the prognostic significance of LNMM of patients with NSCLC has remained highly undetermined. Therefore, we pooled the analysis of all documents that indicated a strong evidence of the independent, adverse prognostic impact of LNMM regarding OS and DFS at the time of the initial diagnosis of operable NSCLC.

Micrometastases disease is the significant cause of mortality from solid tumors. Tumor cells that are detected molecularly in the LN of patients with NSCLC are subjected to one of three fates: death, dormancy, or proliferation [18]. Most metastatic cells die in this hostile microenvironment [19, 20], or they balance the growth rates with the death rates [21]. Other cells adapt by responding to stimuli from the microenvironment.

The poor prognostic significance of LNMM regarding OS and DFS likely occurs because the tumor cells that survive in the LN can completely escape dormancy and begin to proliferate as soon as they reach their destination. Alternately, some of the tumor cells begin to proliferate later after an unknown situation that permits them to overcome inhibitory effects at the metastatic site [18]. Therefore, these tumor cells become viable micrometastases rather than shed tumor cells with a limited life span. Lymph node micrometastasis, as well as overt lymph node metastasis, does not necessarily reflect lymphogenous spread, but it can signal the early phase of hematogenous systemic tumor cell dissemination.

This meta-analysis has shown that the detection of lymph node occult micrometastatic tumor cells is helpful in predicting recurrence and the prognosis of patients with early stage NSCLC. It would also be possible to select patients who might benefit from adjuvant chemotherapy., postoperative adjuvant chemotherapy is not a routine standard therapy for patients with completely resected NSCLC because of its unreliable results for the improvement of prognosis. However, numerous trials, including two randomized trials [22, 23], of induction chemotherapy for stage 3A NSCLC have shown that it was feasible and indicated higher response rates and a survival advantage. Additionally, LNMM patients with a minimal amount of residual tumor could respond better to chemotherapy.

This study has limitations because of the sample size. Although we contacted each author to supplement the missing data, the number of included studies was insufficient to perform some of the preplanned subgroup analyses, and only 3 subgroup studies were included in our analysis. Consequently, we could not identify the effect modifiers that could be attributable to the low statistical power.

## Conclusions

In conclusion, there is evidence that molecular tumor cell detection in regional lymph nodes indicates poor prognosis in node-negative NSCLC. Despite a prognostic impact of tumor cell detection across the applied detection assays, standardized protocols should be used in future studies that use standardized end points and adhere to the UICC definition of tumor cells and micrometastasis. By using such protocols, it should be clarified within prospective randomized trials whether patients with occult lymph node disease benefit from adjuvant chemotherapy. Furthermore, the findings of this study encourage efforts to identify subpopulations of disseminated cells that put patients at a particular risk of disease recurrence and poor survival.
